# Platelet aggregation induced by polystyrene and platinum nanoparticles is dependent on surface area

**DOI:** 10.1039/c8ra07315e

**Published:** 2018-11-12

**Authors:** Fatima Zia, Michaela Kendall, Steve P. Watson, Paula M. Mendes

**Affiliations:** School of Chemical Engineering, College of Engineering and Physical Sciences, University of Birmingham B15 2TT UK p.m.mendes@bham.ac.uk; Centre of Membrane Proteins and Receptors (COMPARE), Universities of Birmingham and Nottingham The Midlands UK; Adelan/School of Engineering, Aston University Birmingham B4 7ET UK http://www.adelan.co.uk; Institute of Cardiovascular Sciences, College of Medical and Dental Sciences, University of Birmingham B15 2TT UK S.P.Watson@bham.ac.uk

## Abstract

Nanoparticles are key components underlying recent technological advances in various industrial and medical fields, and thus understanding their mode of interaction with biological systems is essential. However, while several nanoparticle systems have been shown to interact with blood platelets, many questions remain concerning the mechanisms of platelet activation and the role that the physicochemical properties of nanoparticles play in inducing platelet aggregation. Here, using negatively charged polystyrene nanoparticles with sizes of 25, 50, 119, 151, 201 nm and negatively charged platinum nanoparticles with sizes of 7 and 73 nm, we show that it is not the size of the nanoparticles but rather the nanoparticle surface area that is critical in mediating the effects on platelet activation. The nanoparticles stimulate platelet aggregation through passive (agglutination) and activation of integrin αIIbβ3 through a pathway regulated by Src and Syk tyrosine kinase.

## Abstract 2

Nanoparticles are key components underlying recent technological advances in various industrial and medical fields, and thus understanding their mode of interaction with biological systems is essential.

## Graphical abstract

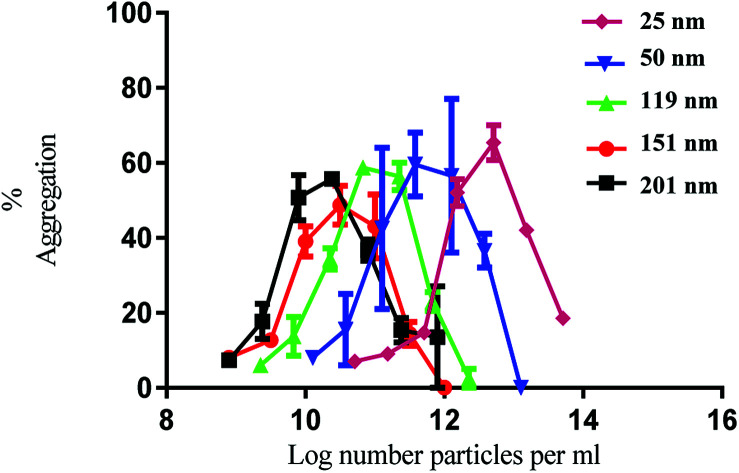

## Introduction

Nanoparticles can be tailored with a diverse range of distinctive physicochemical properties, inducing a plethora of effects upon interaction with biological systems.^1,2^ They can have beneficial, therapeutic or adverse biological effects depending on their characteristics. In this context, the interaction of nanoparticles with platelets may contribute to an undesirable influence on haemostasis or lead to thrombosis. Platelets are central to a healthy vascular system where they maintain haemostasis at sites of injury or inflammation. However, excessive activation of platelets, which leads to their aggregation, is a causative factor for thrombotic diseases that can result in such events as a stroke or heart attack.^3^

Several studies have demonstrated that engineered nanoparticles can interact with platelets in different ways causing either no effect,^4^ inhibition,^5^ and passive (agglutination) or active (integrin αIIbβ3-dependent) aggregation.^6,7^ While the mechanisms and critical factors that drive these effects on platelet function are not yet fully elucidated,^8^ there is increasing evidence that nanoparticle material, shape, size, surface chemistry, surface charge and concentration influence the platelet response. All have been long understood to influence particle mediated health effects.^9^ For instance, multiwalled and single-walled carbon nanotubes,^7^ CdSe quantum dots,^10^ polystyrene nanoparticles^11^ and dendrimers^12^ are able to induce platelet aggregation despite variation in surface charge and hydrophilicity. On the other hand, *in vitro* and *in vivo* studies have shown that either neutral, positively or negatively charged nanoparticles can promote changes in platelet activity, with the degree of activation not only depending on the presence or not of charge but also the nanoparticle size, shape and composition.^11–15^ This intricate relationship between platelet response and nanoparticle characteristics is a result of different mechanisms of platelet activation which depend on the specific interactions of platelet membrane receptors with nanoparticles. The nanoparticle–platelet interaction and response may be mediated either *via* stimulation of platelet surface receptors^10^ or disruption of the platelet membrane.^16^

One important question concerning the behaviour of nanoparticles is whether different nanoparticle sizes can exhibit different functional effects on platelets. A number of studies suggest that small particles might act differently from larger particles.^12,15,17,18^ For instance, exposure of platelets to dendrimers of different generations (G3, G4, G5 and G6) with sizes ranging from 3.1 nm to 7.5 nm led to platelet aggregation only with the larger (G4–G6) dendrimers.^12^ Another study has suggested that the aggregation effect of 20 nm gold nanoparticles was stronger than that caused by 70 nm nanoparticles.^17^ This trend is, to a certain extent, in accordance with a study on silica nanoparticles in which platelet aggregation was observed for 10 nm and not 50 nm nanoparticles.^18^ Since previous size-dependent effect studies have been based on nanoparticle mass and moles concentration as the dose metric, it raises the question on the role that surface area plays in inducing platelet activation. Are the changes in the biological activity of the different size nanoparticles a result of nanoparticle morphology or the greater ratio of surface area and number to mass that occurs as nanoparticle size becomes smaller? Driven by this fundamental question, so critical to understanding the impact of nanoparticles in human health, in this study we investigate the role of nanoparticle surface area mediated effects on platelet activation. Two different nanoparticle materials, namely polystyrene (PS) with sizes of 25, 50, 119, 151, 201 nm and platinum (Pt) with sizes of 7 and 73 nm were used to study active platelet aggregation in a surface area-dependent manner.

## Experimental

### Chemical and materials

Commercially available chemicals were purchased from Aldrich Chemicals and Fisher Chemicals and used as received. Polystyrene nanoparticles were purchased from Polysciences Inc (50 nm; 119 nm; 201 nm) and Bangs Laboratories (25 nm and 151 nm). Dasatinib was from LC-Laboratories; PRT-060318 from Portola Pharmaceuticals and eptifibatide (Integrilin) from GlaxoSmithKline.

### Preparation of Pt nanoparticles

Pt nanoparticles were prepared by adapting the multistep seed-mediated growth protocol reported by Bigall and co-workers.^19^ To begin with, the small (seed) nanoparticles were prepared. A 3.6 ml of a 0.2% (w/v) solution of chloroplatinic acid hexahydrate was added to 46.4 ml of boiling double distilled water. After 1 min of stirring, a 1.1 ml solution of 1% (v/v) sodium citrate and 0.05% (v/v) citric acid solution was added. Half a minute later, a freshly prepared solution (0.55 ml) containing 0.08% (v/v) of sodium borohydride, 1% (v/v) sodium citrate and 0.05% (v/v) citric acid was quickly injected. Following stirring for 10 min, the solution was cooled down to room temperature.

These small nanoparticles act as the templates for the growth of the larger nanoparticles in further reaction steps. To 50 ml of water, 100 μl of nanoparticle seeds prepared as described above and 450 μl of chloroplatinic acid hexahydrate (0.4 M) were added and mixed at room temperature. To this solution, 5 ml of reducing agent (1.25% (v/v) ascorbic acid and 1% (v/v) sodium citrate) was added dropwise, after which the mixture was heated to boiling and left to react under stirring for 45 min. The product thus obtained was purified by centrifugation (30 min, 444*g*) using acetone as the precipitation solvent. Following this, the product was re-dispersed using a mixture of 1 : 4 v/v solution of acetone : water before centrifuging again. This process was repeated 2 times.

### Transmission electron microscopy

A drop of the nanoparticle solution (10 μl) was deposited onto a carbon coated TEM specimen grid (300 mesh, Agar Scientific) and Bright-field imaging was performed using a JEOL 1200ex LaB6 TEM at an accelerating voltage of 80 kV. Particle size distributions were calculated from TEM images using the software ImageJ. The diameters of 200 individual particles were measured to attain the average nanoparticle size from which the standard deviation and frequency size distribution graphs were generated.

### Zeta potential

The zeta potential was measured using a Zetasizer instrument (Malvern Nanosizer Nano ZS) equipped with a red laser light source (HeNe laser operating at *λ* = 632.8 nm) used at 173° backscatter detection mode. The nanoparticles were suspended in the synthesis solvent (double distilled water) or Tyrode's-HEPES buffer (pH 7.3; 134 mM NaCl, 2.90 mM KCl, 0.34 mM Na_2_HPO_4_ : 12H_2_O, 12 mM NaHCO_3_, 20 mM HEPES, 1 mM MgCl_2_ and 10 mM glucose) and were sonicated prior to measurements. Samples were equilibrated at 25 °C for 5 minutes before the measurement. Each sample was measured 3 times (10 measurements each) using disposable capillary cells.

### Preparation of human platelets

Blood was collected from consenting healthy, drug-free volunteers on the day of experiments with 3.8% (w/v) sodium citrate making up 10% of the final volume or 3.2% (w/v) sodium citrate for experiments monitoring aggregation in platelet rich plasma (PRP). Ethical approval for donation of blood by volunteers was granted by Birmingham University Internal Ethical Review (ERN_11-0175). After blood collection, acid-citrate-dextrose (ACD, 10% (v/v)) was added as anti-coagulant. PRP was obtained from anti-coagulated whole blood by centrifuging for 20 min at 200*g* at room temperature. For the preparation of washed platelets, the PRP was further centrifuged at 1000*g* for 10 min at room temperature in the presence of prostacyclin (10 μg ml^−1^) to inhibit platelet activation. The supernatant (plasma) was discarded and the pellet was resuspended in Tyrode's-HEPES buffer. A further washing step was carried out following addition of prostacyclin (10 μg ml^−1^) by centrifuging for 10 min at 1000*g*. The pellet was re-suspended in Tyrode's-HEPES buffer and the washed platelet concentration was adjusted to obtain 2 × 10^8^/ml of washed platelets. Platelets were left for 30 min at room temperature prior to aggregation experiments.

### Platelet aggregation experiments

Platelets (2 × 10^8^/ml) were pre-warmed for 3 min before addition to a Born-aggregometer (ChronoLog). They were then incubated for 2 min with stirring (1200 rpm) prior to nanoparticle addition. Aggregation was recorded for up to 6 min with continual stirring. The final suspension volume was 500 μl. Each particle size was evaluated at different concentrations (surface area per ml), ranging from 1 cm^2^ ml^−1^ to 10^4^ cm^2^ ml^−1^. The surface area was calculated by considering nanoparticle surface area and initial nanoparticle concentration in number of nanoparticles per ml. For the inhibitory studies, the following inhibitors were given 2 min prior to the agonist: eptifibatide (9 μM), dasatinib (10 μM) and PRT-060318 (10 μM). Experiments were carried out three times and error bars are based on standard error of the mean. Data was analysed using GraphPrism and statistical significance was determined using a one-way anova test; differences were considered significant at *P* value < 0.05.

## Results and discussion

Five different sizes of PS particles displaying similar surface chemistry were obtained from two different suppliers. The spherical nanoparticles display diameter sizes ranging from 25 to 201 nm with a narrow size distribution as characterised by TEM and described in Table 1. The PS nanoparticles are unmodified (*i.e.* no surface treatment following nanoparticle preparation) and incorporate sulphate groups on their surfaces as stated by the manufacturers. In order to understand the surface charge density and stability of the nanoparticles and determine how these changes upon exposure to the biological media used to suspend platelets, the zeta potential of the PS nanoparticles in water and Tyrode's-HEPES buffer was measured (Table 1). Tyrode's-HEPES buffer provides osmotic buffering capacity for platelet cells. Zeta potential measurements revealed that all nanoparticles were negatively charged, arising from the presence of the sulphate groups on the surface of the PS nanoparticles. In Tyrode's-HEPES buffer, the zeta potential of the nanoparticles was less negative than in water due to the compression of the electrical double layer at the high ionic strength of the Tyrode's-HEPES medium.^20^ However, all the nanoparticles exhibit high zeta potential values, ensuring the stability of the nanoparticle suspension and prevention of aggregation.

**Table 1:** Average particle size as determined by TEM analysis and zeta potential of the PS nanoparticles in water (pH = 6.8) and Tyrode's-HEPES buffer (pH = 7.3)

Nanoparticle	Average particle size + SD (nm)	Zeta potential ± SD (mV)	
		Water	Tyrode's-HEPES buffer
PS-25	25.2 ± 4.9	−42.5 ± 2.0	−39.4 ± 1.9
PS-50	50.4 ± 4.9	−50.8 ± 1.6	−35.8 ± 1.6
PS-119	119.2 ± 8.5	−35.9 ± 0.7	−31.7 ± 1.3
PS-151	150.6 ± 9.7	−46.1 ± 1.1	−25.2 ± 1.8
PS-201	200.7 ± 10.7	−56.3 ± 1.2	−33.7 ± 1.4

**Fig. 1:**
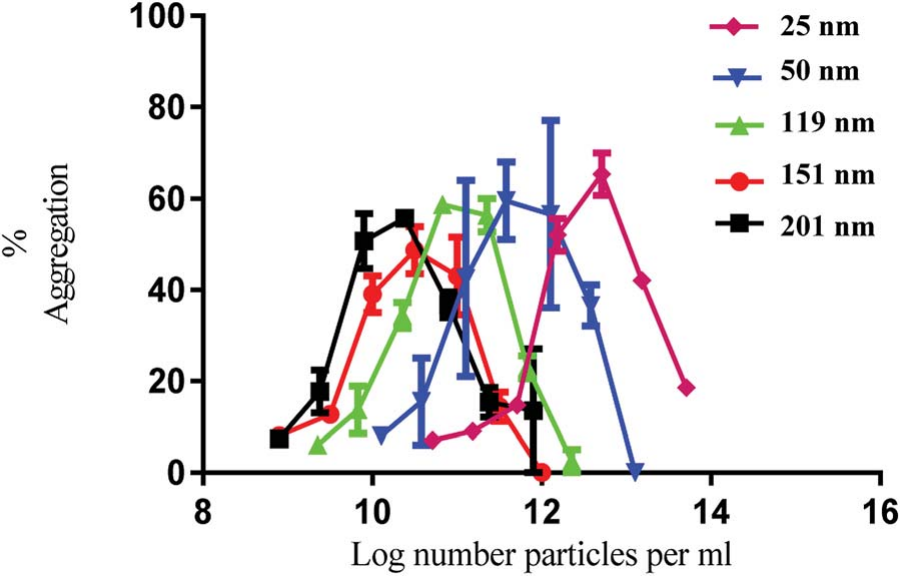
Nanoparticle induced platelet aggregation in response to various sizes of polystyrene particles. The % aggregation represents the difference in light transmission between the platelet suspension and a clear solution. The data is from 3 experiments and shown as mean + SEM.

Having established the physicochemical characteristics of the PS nanoparticles, the effect of size on the functional activity of human platelets *in vitro* was investigated. Human washed platelets were stimulated with PS nanoparticles and monitored using light transmission aggregometry (LTA). All the nanoparticles induced concentration-dependent platelet aggregation indicated by the increase in light transmission (Fig. 1). There was no difference in the maximal level of aggregation which was similar to that induced by strong platelet agonists such as collagen and thrombin.^21^

**Fig. 2:**
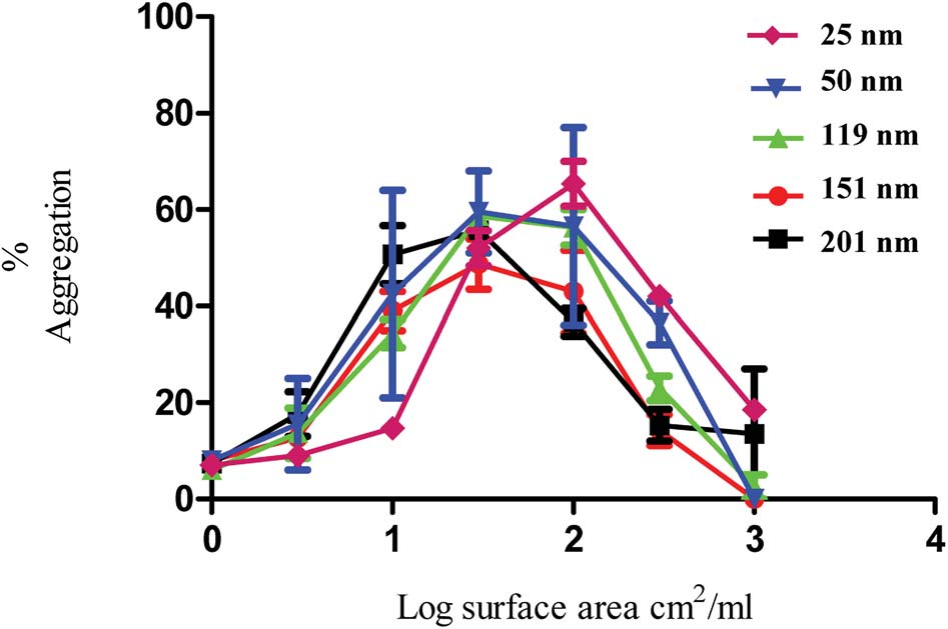
Nanoparticle induced platelet aggregation in response to various sizes of PS nanoparticles. The data on the *x* axis is shown as log surface area (cm^2^ ml^−1^). The % aggregation represents the difference in light transmission between the platelet suspension and a clear solution. The data is from 3 experiments and shown as mean + SEM.

The nanoparticles exhibited bell-shaped dose response curves. Maximum aggregation levels feature at different particle concentrations, with the larger nanoparticles giving rise to significant aggregation at lower nanoparticle concentrations. Importantly, while these results seem supportive of a trend between nanoparticle size and their potency in causing platelet aggregation, they do not take into account the nanoparticle surface area. By considering surface area as a significant nanoparticle characteristic, the data shown in Fig. 1 was re-plotted to illustrate the relationship between surface area and aggregation (Fig. 2).

As demonstrated in Fig. 2, the re-plotted data produced similar bell shaped curves, in which all nanoparticle size trends were super-imposable. These findings imply that the surface area of the PS nanoparticles is a critical factor in inducing platelet activation, independently of nanoparticle size. Thus, if one considers surface area as the most relevant dose metric, higher nanoparticle concentrations of the smallest nanoparticles are required to reach similar impact in platelet aggregation as the largest ones, as supported by the trends shown in Fig. 1. These results thus highlight not only the importance of assessing platelet activity in different dose metrics to understand nanoparticle characteristic effects but also the predominant role of surface area towards platelet activation.

**Fig. 3:**
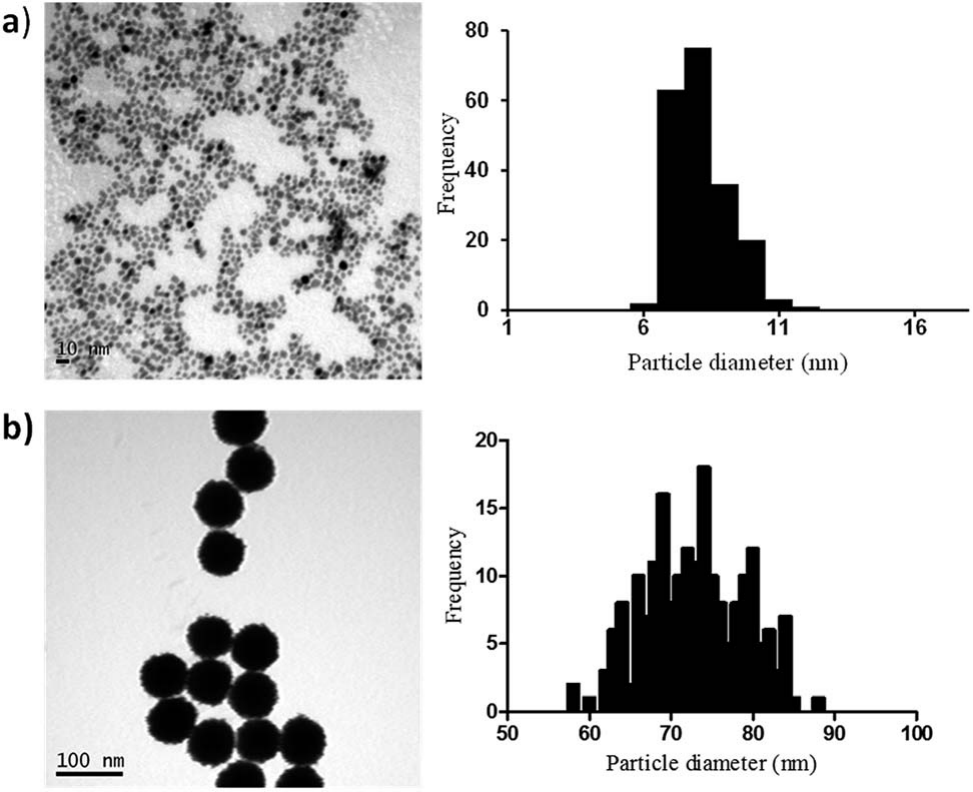
TEM images and particle size distributions for the synthesised Pt nanoparticles with mean diameter + SD of (a) 7.1 ± 1.1 nm and (b) 72.9 ± 6.0 nm.

In order to elucidate if similar surface area-dependent trend appears for other nanoparticle types, two different sizes Pt nanoparticles (*i.e.* 7 nm and 73 nm) were prepared and characterised. Small Pt nanoparticles (7 nm) were synthesized using a sodium borohydride reduction method and these Pt nanoparticles acted as the seeds for the synthesis of larger Pt nanoparticles (73 nm) using ascorbic acid as the reducing agent. Sodium citrate and citric acid were used as surfactants to stabilise the nanoparticles, leading to the formation of nanoparticles coated with citrate ions and citric acid. TEM indicates the formation of monodispersed nanoparticles with spherical shape and narrow size distribution, with small Pt nanoparticles having an average diameter of 7.1 ± 1.1 nm and the larger Pt nanoparticles of 72.9 ± 6.0 nm (Fig. 3).

Zeta potential values for the small and large Pt nanoparticles when dispersed in both water and Tyrode's-HEPES buffer are reported in Table 2. As expected, the nanoparticles are all negatively charged due the adsorbed monolayers of citrate and citric acid on their surfaces. Within the same medium, the small and large nanoparticles have comparable zeta potential values. In a similar manner as for the PS nanoparticles, the zeta potential values obtained for Tyrode's-HEPES buffer are lower than those in water. However, in both media, the nanoparticles have limited tendency to aggregate since they are highly stable in water (zeta potential ranges between −52.3 to −59.3 mV) and moderately stable in Tyrode's-HEPES medium (zeta potential ranges between −20.8 to −22.4 mV).

**Table 2:** Zeta potential of the Pt nanoparticles in water (pH = 6.8) and Tyrode's-HEPES buffer (pH = 7.3)

Nanoparticle	Zeta potential ± SD (mV)	
	Water	Tyrode's-HEPES buffer
Pt-7	−59.3 ± 3.2	−20.8 ± 0.8
Pt-73	−52.3 ± 0.8	−22.4 ± 1.3

**Fig. 4:**
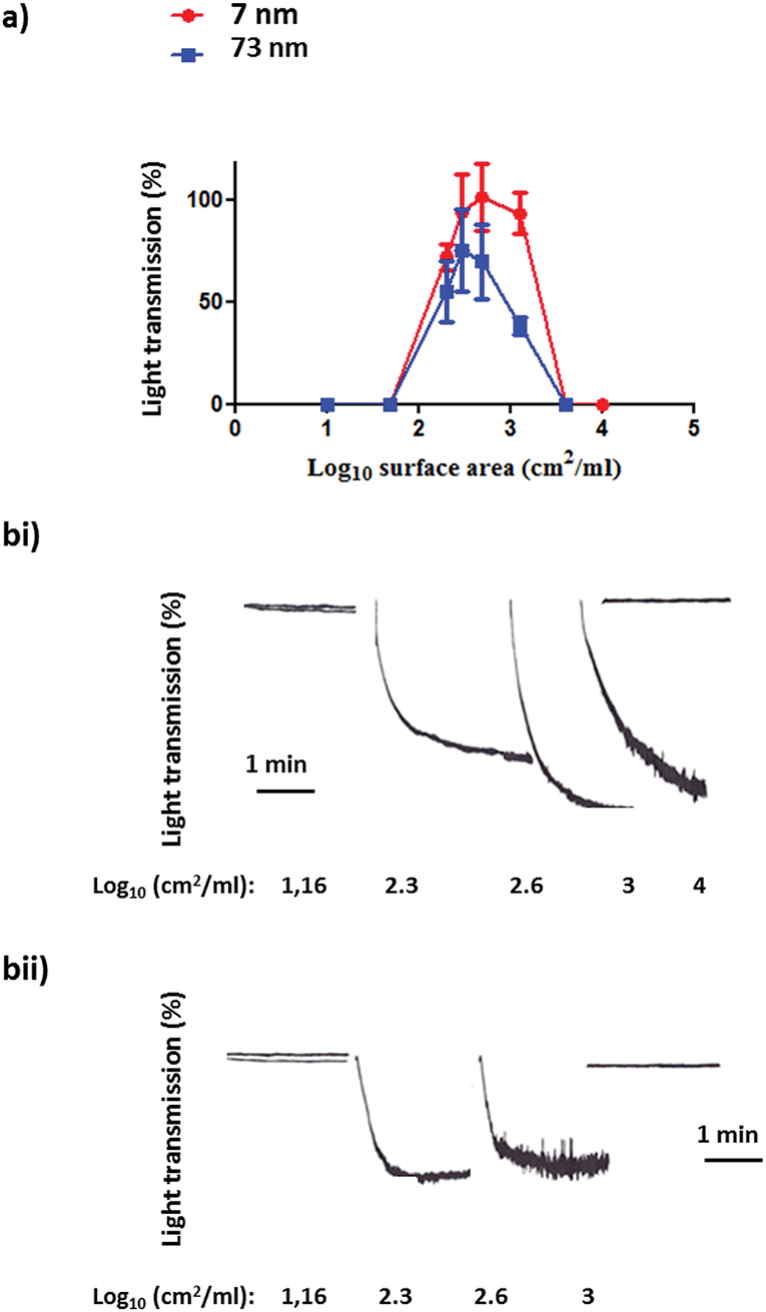
(a) Surface area-based dose response curve for platelet aggregation to platinum nanoparticles (log_10_ 1–4 cm^2^ ml^−1^). The results as shown as the % of the maximal aggregation response. The data is from 3 experiments and shown as mean + SEM. (b) Representative aggregation traces to (i) 7 nm and (ii) 73 nm platinum nanoparticles.

**Fig. 5:**
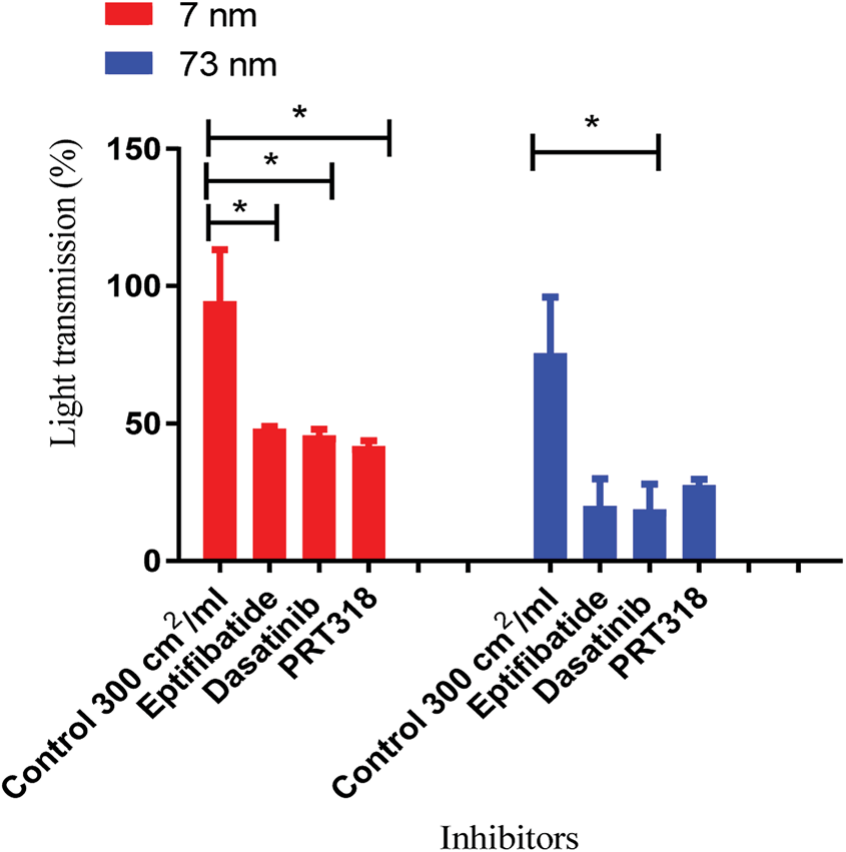
Mechanism of aggregation by platinum nanoparticles. Nanoparticle controls were dosed at log_10_ 2.4 cm^2^ ml^−1^ as this was the surface area that induced maximal aggregation. The concentration of washed platelets was 2 × 10^8^/ml. Platelets were preincubated with integrilin (9 μM), dasatinib (10 μM), PRT-060318 (10 μM) or vehicle (DMSO). Data is the mean + SEM of three experiments. Statistical analysis is based on a one-way anova and a Tukey test, where (**P < 0.01).

Following the characterization of the Pt nanoparticles, attention was turned to the investigation of the effect of surface area on the aggregation of human platelets. Fig. 4(a and b(i–ii)) shows the nanoparticles surface area-based dose response curve for the 7 nm and 73 nm Pt nanoparticles (log_10_ 1–4 cm^2^ ml^−1^). Both particles stimulated rapid aggregation without a noticeable preceding shape change (which manifests as a decrease in light transmission) and produced a similar bell shaped curve, with no measureable aggregation at high doses. Platelet aggregation was observed in the middle of the dose range used. The Pt nanoparticles caused a similar maximal increase in light transmission to that of the PS nanoparticles and this was not statistically different between the two sized particles (Fig. 5).

In order to place the observations above into a physiological context of exposure, Pt nanoparticles were tested in platelet rich plasma (PRP). PRP is highly enriched in clotting factors, growth factors and proteins such as albumin, fibrinogen and globulins. The results in PRP showed that the platinum nanoparticles (7 nm and 73 nm) did not cause aggregation of platelets (not shown). Collagen was used as a positive control in which a typical collagen response was seen, *i.e.* shape change followed by aggregation (not shown). This result is likely to reflect non-specific binding of platinum nanoparticles to plasma proteins.^22–24^

To ascertain whether the response to nanoparticle stimulation in washed platelets was mediated by integrin αIIbβ3-dependent aggregation, inhibitory studies were performed using the αIIbβ3 receptor antagonist Integrilin and the Src and Syk tyrosine kinase inhibitors dasatinib and PRT-060318, respectively. The rational for these experiments is that the use of Integrilin will establish whether this is passive (agglutination) or receptor driven aggregation, while the two tyrosine kinase inhibitors will provide information on the mechanism of aggregation. Src and Syk tyrosine kinases mediate activation of platelets by a group of single transmembrane receptors which signal through an immunoreceptor-tyrosine-based-activation-motif (ITAM) namely GPVI, CLEC-2 and FcγRIIA. These three receptors are activated by clustering which is the likely mechanism of activation by the polyvalent nanoparticles.

Prior to the stimulation of platelets by nanoparticles, the platelets were incubated separately with the inhibitors for 2 min. The effect of the inhibitor was compared to vehicle-treated (DMSO) controls. The dose that produced maximal platelet aggregation for both sizes (log_10_ 2.4 cm^2^ ml^−1^) of nanoparticle was used.

All three inhibitors caused a partial (50–70%) inhibition of platelet activation to both sizes of Pt nanoparticles (Fig. 5). The partial inhibition by Integrilin demonstrates that aggregation is composed of two phases, agglutination (which is not blocked by Integrilin) and integrin αIIbβ3-mediated aggregation (which is blocked by Integrilin). The similar inhibitory effect of dasatinib and PRT-060318 demonstrates that activation of integrin αIIbβ3 is likely to be mediated by one or more of the three ITAM receptors described above. The mechanism of agglutination could be through neutralisation of platelet surface charge leading to binding of membrane proteins to each other. Interestingly, a similar profile of results has been reported for activation of human and mouse platelets by diesel exhaust particles, with activation mediated by GPVI with a minor contribution of CLEC-2.^25^

## Conclusions

In conclusion, the present study shows that the activation of platelets by two types of nanoparticles, both of which have an overall negative charge, is governed by their surface area and not the size, and that the dose response curve for activation is bell shaped. Activation of platelets is mediated by passive agglutination and activation of integrin αIIbβ3 through a pathway regulated by Src and Syk tyrosine kinase (likely through crosslinking of GPVI and CLEC-2). The relationship between nanoparticle surface area and platelet activation, irrespective of nanoparticle surface type, is consistent with crosslinking of surface receptors being a key determinant in inducing platelet activation. The bell-shaped nature of the dose response curve could reflect repelling of platelets from each due to the charge of the particles. The results have important implication for the design of nanoparticles in targeting surface receptors and understanding surface interactions of platelets with foreign bodies.

## Conflicts of interest

There are no conflicts to declare.

## Supplementary Material
